# Supplementary material for: A Single Dose of PEG-Asparaginase at the Beginning of Induction Not Only Accelerates MRD Clearance but Also Improves Long-Term Outcome in Children with B-Lineage ALL
Source: Cancers (Basel). 2023 Nov 23;15(23):5547. doi: 10.3390/cancers15235547 (PMC10705323; doi:10.3390/cancers15235547)
Supplement: Supplementary file 1 [file cancers-15-05547-s001.zip › cancers-2632737-supplementary.pdf]

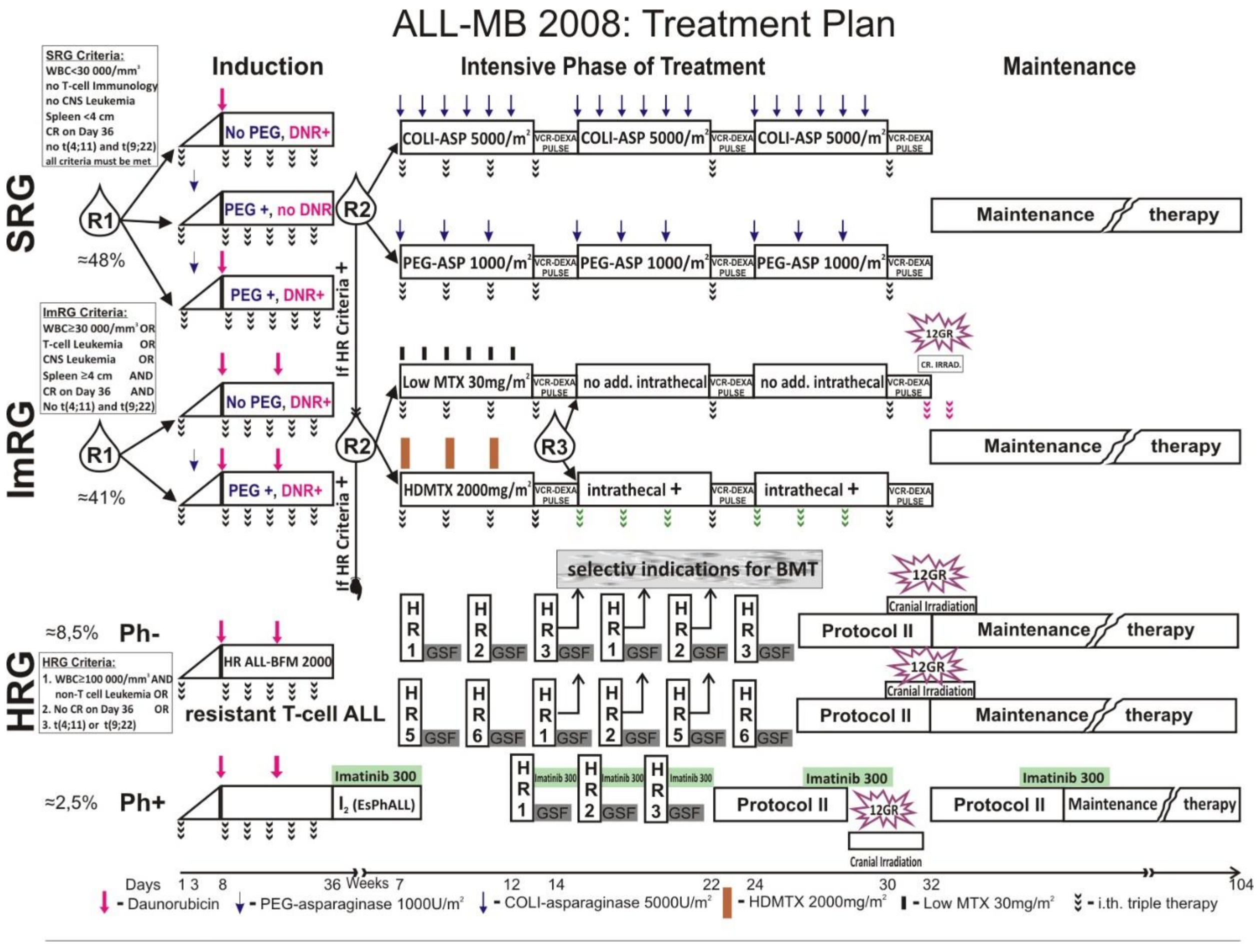

Figure S1. Protocol ALL-MB 2008: Overview

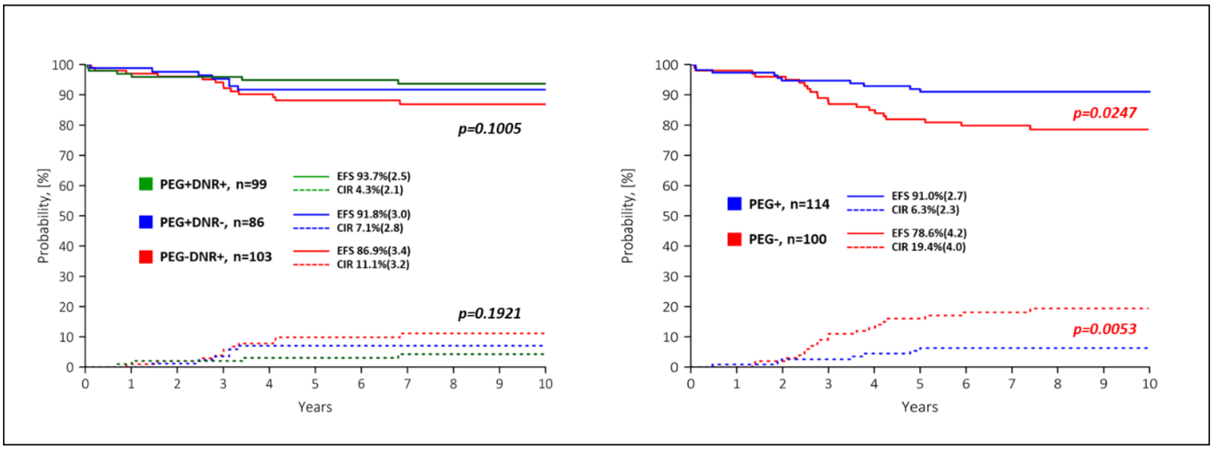

Figure S2. Event-free survival (EFS, solid lines) and cumulative incidence of relapse (CIR, dashed lines) in patients enrolled in the MRD study and randomized in induction for various regimens in respect to day 3 PEG-asparaginase (PEG) administration. The left panel shows patients of the SR group (n=288), while right panel shows patients of the ImR group (n=214). DNR – Daunorubicine
